# Optimizing Individual Wound Closure Practice Using Augmented Reality: A Randomized Controlled Study

**DOI:** 10.7759/cureus.59296

**Published:** 2024-04-29

**Authors:** Marissa Lovett, Eze Ahanonu, Allyson Molzahn, David Biffar, Allan Hamilton

**Affiliations:** 1 Department of Health Sciences, University of Arizona, Tucson, USA; 2 Department of Electrical and Computer Engineering, University of Arizona, Tucson, USA; 3 Department of Surgery, University of Arizona, Tucson, USA

**Keywords:** virtual augmented reality, suture skill acquisition, medical education technology, augmented reality (ar), simulation in medical education

## Abstract

Background

Suturing requires repeated practice with guidance to prevent skill deterioration; however, guidance is often limited by expert availability. There is evidence that augmented reality (AR) may assist procedural skill acquisition among learners. This study examines the use of an AR suture guidance application to assist the independent practice of suturing.

Methodology

A novel suture guidance application was designed for the Microsoft HoloLens. The guidance system included a calibration system and holograms that projected over a suture pad in a stepwise manner. To assess the application, 30 medical students were recruited and randomly assigned to two groups. The control group (n = 16) was given 30 minutes of independent suture practice, while the experimental group (n = 14) utilized the suture guidance application. Both groups completed a pre- and post-test wound closure assessment. After the post-test, the control group trialed the suture guidance application. All participants completed a feedback survey on the application. Statistical analysis was completed using Stata (StataCorp., College Station, TX, USA) with paired Student’s t-tests and Welch’s t-tests with a significance of 95%.

Results

Both groups demonstrated a significant improvement in total time and time per stitch during the post-test. Additionally, comparing pre- and post-test assessments in the experimental group revealed a significant improvement in the total number of stitches (p = 0.007), the ratio of bisecting stitches (p = 0.02), and the symmetry of stitch bite (p = 0.03). The feedback survey supported the application for guiding suture placement and spacing. Participants identified limitations in the hologram stability and neck positioning.

Conclusions

This study suggests the potential to use AR to facilitate the independent practice of wound closure within simulation environments.

## Introduction

Previous literature has shown that medical students entering residency reported the greatest anxiety regarding performing basic procedural skills [1]. Similarly, medical students reported experiencing significant barriers to practicing suture skills, with the most common obstacle being a lack of opportunity [2]. As interns across specialties are expected to apply clinical skills independently within the first months of their residency, there is a greater need for medical students to solidify these skills before entering residency programs [3]. Moreover, despite instructor-led training courses that teach procedural skills, these competencies deteriorate over time [4,5]. Many factors contribute to the decay of procedural skills after acquisition, including lack of repeated practice and/or lack of feedback during individual practice [5]. Although direct expert feedback remains the gold standard in simulation-based procedural education, instructors are often not readily available to provide the kind of guidance that medical students need to refine their technical skills over time [6]. Once a medical student has completed the initial skill acquisition via expert instruction on how to properly suture, students may benefit from a technology that can assist in minimizing drift from initial instruction and aid in refining skills. Augmented reality (AR) is a rapidly developing technology that can be used after live expert instruction by providing guidance during self-directed learning to assist with the maintenance of technical skill proficiency [7,8].

The Microsoft HoloLens AR technology overlays virtual projections on top of the learner’s natural environment using a head-mounted display system [9]. AR applications have already proven to be advantageous in anatomy, laparoscopic surgery education, as well as the clinical care of patients [7,10-14]. In certain applications, AR environments can be advantageous compared to virtual reality (VR), where learners interact in a fully immersive virtual environment. Comparative advantages of AR technology include fewer negative user impacts and greater realism and interaction with the learner’s natural environment [8,15-18]. While the initial investment in AR technology may be significant, it entails minimal ongoing maintenance and operational costs [15].

In this study, a Microsoft HoloLens application was designed to act as a guidance system to refine suturing skills among medical students. Previous studies implementing AR technology into laparoscopic suture training have successfully led to suturing skill acquisition and limited critical errors for medical and pre-medical students [13,19]. AR-guided suturing systems also have the capability to provide real-time and summative feedback for students practicing suturing skills [20]. These studies provided a framework for a new application designed to guide wound closure. This study aimed to design and implement an AR-guided simple suture application. It sought to then evaluate change in performance closing a simple laceration after training with the AR-guided simple suture application compared to independent practice.

## Materials and methods

Development of a simple laceration model

The simple laceration model used was produced in-house using a silicone base, a memory foam layer to represent soft tissue, a mesh layer for the durability of the model, and a silicone top layer to represent skin. A 7.5 cm laceration that extended into the memory foam layer was cut down the center of the model for wound closure. The laceration model was secured in a plastic base that was suctioned to the table.

Development of a wound closure application for Microsoft HoloLens

This application was developed using the Unity Game Engine (Unity Technologies, 2018), a development platform for creating games and interactive simulations. The application was designed to allow for an interactive, guided practice of wound closure. To achieve this, the placement of holograms was optimized to improve display accuracy and stability at distances below the 2-m focal plane suggested by Microsoft for standard HoloLens applications. Three-dimensional (3D) modeling of holograms for simple laceration closure was designed using Autodesk Inventor 2019 (Autodesk, 2018), a 3D modeling software for modeling and simulation, and imported into the Unity environment.

**Figure 1:**
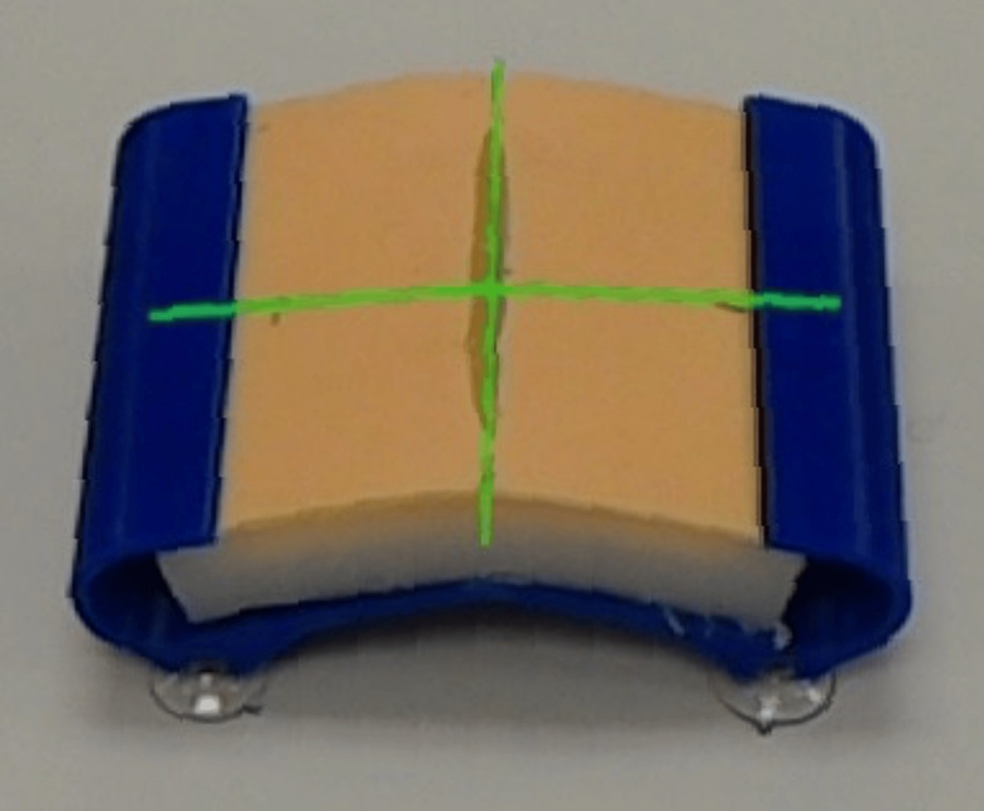
Suture pad calibration. Alignment of the simple laceration model with holographic calibration guide. This image was captured through the HoloLens and represents what is seen by participants through the HoloLens.

**Figure 2:**
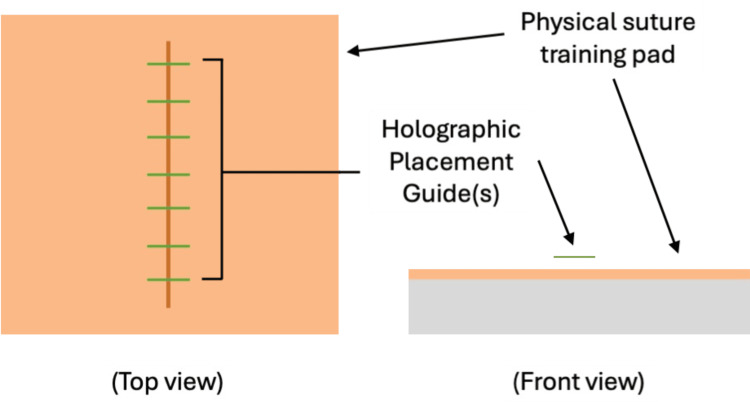
Illustration of holographic guidelines. Holographic guidelines are projected on top of a simple laceration model.

**Figure 3:**
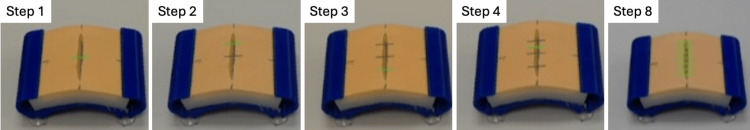
Progressing guidance of suture placement. Images depicting the progressing guidance of suture placement. Above are pictures of the first four steps and the last step, Step 8, in the suturing guidance system. These images were captured through the HoloLens and represent what is seen by participants through the HoloLens. Suggested suture placement is indicated by green lines. Sutures placed by the learner in previous steps are indicated by black lines.

Wound closure objectives reinforced with this application were the order of stitch placement, appropriate spacing between stitches, appropriate distance from the wound edge, and symmetrical distance from the wound edge. To meet these objectives, the application used audio and visual cues to direct the learner through calibration (Figure 1), projected lines over the wound to guide wound closure (Figure 2), and used learner commands to progress through the guidance system in a stepwise manner (Figure 3). Calibration was required to ensure the proper function of sensors and the correct initial setup.

**Figure 4:**
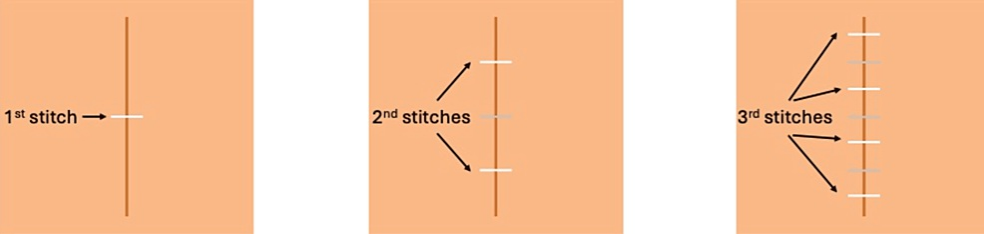
Illustration of the stitch order using the “Rule of Halves.”

The order of guideline placement followed the “Rule of Halves” which is preferred for simple interrupted suture placement to improve wound edge alignment (Figure 4) [21]. Each guideline, depicted in green in Figure 3, was 8 mm in length with 10 mm spacing between adjacent stitches. Sutures placed by the learner in previous steps turned black as they progressed through the guideline placement.

Study protocol

The Institutional Review Board of the University of Arizona approved this study on March 8, 2011 (ID: 1100000081). Written informed consent for inclusion in this research was obtained before enrollment in the study. A power analysis was completed with an α of 0.05 and an effect size of 0.5 to estimate a sample size of 32. A total of 30 medical students were recruited for this study at the Arizona Simulation Technology and Education Center (ASTEC). The participants were randomly assigned into one of two groups, a control group (n = 16) and an experimental group (n = 14). Before the study, a random number generator was used to assign participants a numerical identifier. Participants were randomly stratified based on reported hours of suture experience to either the control or experimental group. The primary researcher generated the random allocation sequence, enrolled participants, and assigned participants to either the experimental or control intervention. Eligible participants were medical students. The study was conducted within a dedicated research space at ASTEC.

All participants viewed an introductory video on wound closure [21]. All participants then completed a suturing pre-test that consisted of closing a 7.5 cm straight laceration using a simple interrupted suturing technique with a 2-0 silk suture. Participants were given unlimited time and suture to complete this task. Each participant was also provided suturing instruments, i.e., a needle holder, scissors, and forceps.

After the pre-test, the control group was given a half hour to practice suturing skills independently. The experimental group was given a half hour to practice suturing using the AR wound closure application. A half hour was chosen as it approximates the average amount of time medical students practice suturing independently at ASTEC. Participants within each group then completed a suturing post-test that replicated the pre-test conditions.

The pre- and post-tests were assessed on the total number of stitches, the ratio of stitches that bisected the wound, the total wound closure time, the symmetry of stitch bite, the width of the stitch, the angle of the stitches, and the spacing between stitches. A stitch that bisects the wound refers to a technique where the thread is passed through the tissue of a wound in a way that divides it into two parts, helping to close it securely. Data for the position of the first stitch, the total number of stitches, the ratio of stitches that bisected the wound, and the total wound closure time were collected by the researcher during the study through direct observation and the use of a stopwatch. After the study was completed, data were collected on the symmetry of the stitch bite, the width of the stitch, the angle of the stitches, and the spacing between stitches from the pre- and post-test suture pads. The width of the stitch was measured by a ruler. The symmetry of the stitch bite was calculated as a ratio of the bite width to the left of the wound over the bite width to the right of the wound. The angle of stitches was measured with a protractor as degrees above or below perpendicular to the wound edge.

After the post-test, the control group was given the opportunity to practice suturing while using the AR wound closure application. All participants completed a survey using a five-point Likert scale to provide feedback on the wound closure application.

Statistical methods

The collected data were compiled within a Microsoft Excel spreadsheet (Microsoft Corp., Redmond, WA, USA) and all statistical analysis was performed in Stata, a statistical software (StataCorp., College Station, TX, USA). Paired Student’s t-tests with a significance of 95% were used to compare pre- and post-test results within the control and experimental groups. Statistical comparisons between experimental and control groups were obtained using a Welch’s t-test with a significance of 95%.

## Results

Demographic data were obtained from all participants to assess prior experience with suturing and HoloLens use (Table 1). There was no significant difference in the number of suture training attended, number of hours practiced, or number of times sutured in clinical practice between the control and experimental group participants. Both groups had no prior experience using Microsoft HoloLens. All participants were included in the analyses.

**Table 1:** Demographic data of study participants *: Scored on a five-point Likert Scale: 1 = 0 trainings; 2 = 1-2 trainings; 3 = 3-5 trainings; 4 = 6-9 trainings; 5 = 10+ trainings attended and reported as mean ± standard deviation.

Variable	Control	Experimental
Sample size, n	16	14
Medical student year, n (%)		
I	4 (25)	5 (35.71)
II	6 (37.5)	4 (28.57)
III	3 (18.75)	4 (28.57)
IV	3 (18.75)	1 (7.14)
Number of suture trainings attended^*^	2.5 ± 1.2	2.4 ± 1.2
Number of hours practiced sutures^*^	2.9 ± 1.7	2.8 ± 1.7
Number of times sutured in clinical practice^*^	2.6 ± 1.7	2.4 ± 1.9
Prior augmented reality experience, %	25	21.4
Prior HoloLens experience, %	0	0

Suture data was obtained from the pre- and post-test to assess the total number of stitches, the ratio of stitches that bisected the wound, the total wound closure time, the symmetry of the stitch bite, the width of the stitch, the angle of stitches, and the spacing between stitches (Table 2). There was a significant decrease in the total wound closure time and the time per stitch in both the control and experimental groups when comparing pre- and post-test assessments.

**Table 2:** Comparison of pre- and post-test wound closure assessments. Comparison of pre- and post-test wound closure assessments for the control and experimental groups. This was calculated by subtracting the pre-test from the post-test for each participant. The data were then averaged for each group and expressed as the mean of the difference between pre-test and post-test assessments ± standard deviation. Statistical analysis was performed using a paired t-test. P-values <0.05 were considered significant.

Wound closure categories	Control (pre-post)		Experimental (pre-post)	
	Mean ± SD	P-value	Mean ± SD	P-value
Total number of stitches, ± SD	0.3 ± 1.5	0.26	0.6 ± 0.8	0.007
Ratio of bisecting stitches, ± SD	-0.03 ± 0.2	0.29	-0.1 ± 0.2	0.02
Total wound closure time, seconds, ± SD	188.9 ± 216.9	0.002	274.1 ± 142.9	3.59E-6
Time per stitch, seconds per stitch, ± SD	20.7 ± 23.5	0.002	30.7 ± 22.8	0.0001
Symmetry of stitch bite, ± SD	-0.05 ± 0.2	0.14	0.03 ± 0.05	0.03
Width of stitches, mm, ± SD	-0.04 ± 0.2	0.20	0.1 ± 0.3	0.14
Angle of stitches, degrees, ± SD	1.2 ± 3.4	0.10	1.6 ± 5.4	0.15
Spacing between stitches, mm, ± SD	-0.03 ± 0.2	0.23	-0.05 ± 1.2	0.11

When comparing the post-test wound closure assessments between the experimental group and control group, the experimental group also used significantly fewer stitches (p = 0.021) and the space between stitches was significantly greater (p = 0.031) during the post-test wound closure (Table 3).

**Table 3:** Comparison of control and experimental group performance on the post-test. Comparison of control and experimental post-test wound closure assessments. The data are expressed as the mean of either the control or experimental group’s average post-test assessment ± standard deviation. Statistical analysis was performed using Welch’s t-test. P-values <0.05 were considered significant.

Wound closure categories	Control (post)	Experimental (post)	P-value
Total number of stitches, ± SD	8.4 ± 2.55	6.9 ± 0.62	0.02
Ratio of bisecting stitches, ± SD	0.76 ± 0.35	0.92 ± 0.13	0.05
Total wound closure time, seconds, ± SD	665.6 ± 233.96	581.2 ± 138.29	0.12
Time per stitch, seconds per stitch, ± SD	80.6 ± 24.9	83.8 ± 18.4	0.35
Symmetry of stitch bite, ± SD	0.60 ± 0.19	0.53 ± 0.05	0.07
Width of stitches, mm, ± SD	1.05 ± 0.28	1.07 ± 0.26	0.41
Angle of stitches, degrees, ± SD	5.14 ± 2.49	4.27 ± 1.86	0.141
Spacing between stitches, mm, ± SD	0.90 ± 0.28	1.03 ± 0.26	0.031

The wound closure objectives were also evaluated by all participants in an application feedback survey after the use of the Microsoft HoloLens suturing application (Table 4). Participants agreed the applications were useful for guiding suture placement and spacing between sutures. The application was less successful in guiding the appropriate bite width and symmetrical bite width from the wound edge. There were no significant differences between the control and experimental participant responses.

**Table 4:** Results from HoloLens suturing application survey. Results from the HoloLens suturing application survey for all participants (n=30). The survey was scored on a five-point Likert scale: 1 = strongly disagree; 2 = disagree; 3 = neutral; 4 = agree; and 5 = strongly agree. Data are expressed as mean ± standard deviation.

Suture application objectives	All participants (n = 30)
Useful to guide the order of suture placement	4.2 ± 0.8
Useful to guide the appropriate spacing between sutures	4.2 ± 1.2
Useful to guide the appropriate bite width from the wound edge	3.7 ± 1.2
Useful to guide the symmetrical bite width from the wound edge	3.7 ± 1.1

Participants were also given the opportunity to provide open-ended responses to the clinical utility of this application, the educational utility of this application, and suggestions for future modifications. All participants agreed the suture guidance system is an educationally useful application of AR and that there is a clinical utility with further refinement. Two modifications were suggested by the majority of the participants for application improvement. Specifically, participants identified the need to improve hologram stability and uncomfortable neck positioning as current limitations of the application.

## Discussion

The AR application developed in this work demonstrated a method to allow instructor-led training to be complemented by guided individual practice. As shown in Table 2, the medical students who utilized the suture guidance application showed significant skill improvement in more categories than the students within the control group. Both the experimental and control groups showed improvement in the total wound closure time and the time per stitch. The group that used the suture guidance application also improved the ratio of stitches that bisected the wound, the number of stitches placed to close the wound, and the symmetry of the bite. When comparing post-test performance between groups, the experimental group performed significantly better than the control group based on the total number of stitches and spacing between stitches. These results suggest the use of the AR suturing guidance system may be beneficial for the guided-independent practice of suturing skills at the medical student level.

Since the completion of this study, HoloLens 2 has been released with additional sensors for improved eye and gesture tracking for a smoother experience. Given the unsteadiness noted by participants, employing a suture training program using HoloLens 2 could improve learning outcomes and address some of the limitations of the current study. In a previous study, wound closure procedure time was significantly lower in novice medical students who practiced using an AR program compared to an instructional video [22]. Previous studies comparing scoring assessments of wound closure by experts and an AR suturing program showed AR was a valid tool for assessing laparoscopic suturing performance [20]. This suggests AR could be used to both independently practice and receive accurate assessments on performance, a resource that would allow novice medical students to continue to grow their suturing skills after initial acquisition. Although advanced AR technologies are promising, the rapid growth of extended reality technologies has led to the introduction of mixed reality technologies, which serve to combine AR and VR to create an immersive realistic scenario [23]. This mixed reality technology is anticipated to be incorporated more frequently into extended reality programs in medical education. This foundational data using AR should be explored using XR-compatible technologies.

Limitations

This study has some limitations. First, the sample size is smaller than desired. A power analysis was conducted before starting this study, which indicated a sample size of 32 would be sufficient to detect the effects hypothesized. Due to time constraints, only 30 participants were recruited for this study. While this falls slightly below the calculated sample size, this is a preliminary study, and these findings offer valuable insights to be used for future studies. In the future, the sample size would be increased to ensure the study is sufficiently powered.

Second, participants noted in the feedback survey that the holograms bounced in place. Hologram instability may account for the lack of significant improvement in the angle of stitches, width of bite, and spacing between stitches. Although the hologram movement is less noticeable when projecting large holograms at a distance, it was detrimental when guiding precise distances within arms-length of the participant. Notably, this hologram instability also prevented the initial application design from including depth guidance within the inside of the wound, a crucial criterion of proper suture placement. In addition to lacking guidance on the depth of the suture, the program did not address the economy of motion while placing the sutures. Although the total wound closure time and time per stitch were measured before and after practice, this does not fully capture the efficiency of the wound closure time. Improvements in the hologram stability and AR application in future generations of the Microsoft HoloLens would likely add to the utility of this application in guiding stitch width, symmetry, spacing, depth, and measuring economy of motion.

Additionally, the weight of the HoloLens with the required neck positioning to project the holograms on a suture pad at table height was uncomfortable for participants. Adjustments in table height and suture pad angle were found to optimize the working conditions; however, these conditions are not easily replicated in all learning environments. Enlarging the hologram projection field in future HoloLens generations may add to the usability of the head-mounted display in educational and clinical settings.

Another consideration is the cost and time investment required for the use and operation of an AR suture application. As mentioned previously, the initial cost of AR technology is significant and not feasible for all programs. While there are few ongoing maintenance costs once the initial cost is covered, this should be considered. Lastly, as this was a preliminary study, the program was not designed to adjust to the placement of sutures as they happened live. This means the program did not adjust its guidance based on where a suture was placed by the participant. In future versions of the product, this feature would be included to provide live feedback on the participant’s performance. Additionally, future studies should compare how AR guidance compares to a simple laceration model with guidelines drawn on.

## Conclusions

Overall, this study provides evidence that may suggest AR can be used to guide independent practice after initial skill acquisition of wound closure within simulation environments and guidance for improved utility of AR technology in medical education. Future work will explore the implementation of a similar suture training program using XR technologies, as well as an in-depth study of suture skill retention with and without AR. Additionally, more complex wound closure scenarios will be considered to allow more advanced skills to be trained. This application will likely provide additional educational benefits for future generations of extended reality that address hologram stability, comfort of head-mounted devices, and increased projection field.
